# Exploiting the CRISPR/Cas9 PAM Constraint for Single-Nucleotide Resolution Interventions

**DOI:** 10.1371/journal.pone.0144970

**Published:** 2016-01-20

**Authors:** Yi Li, Saurabh Mendiratta, Kristina Ehrhardt, Neha Kashyap, Michael A. White, Leonidas Bleris

**Affiliations:** 1 Bioengineering Department, The University of Texas at Dallas, 800 West Campbell Road, Richardson, Texas 75080, United States of America; 2 Center for Systems Biology, The University of Texas at Dallas, 800 West Campbell Road, Richardson, Texas 75080, United States of America; 3 Department of Cell Biology, University of Texas Southwestern Medical Center, 5323 Harry Hines Blvd., Dallas, Texas 75390, United States of America; 4 Electrical Engineering Department, The University of Texas at Dallas, 800 West Campbell Road, Richardson, Texas 75080, United States of America; SRI International, UNITED STATES

## Abstract

CRISPR/Cas9 is an enabling RNA-guided technology for genome targeting and engineering. An acute DNA binding constraint of the Cas9 protein is the Protospacer Adjacent Motif (PAM). Here we demonstrate that the PAM requirement can be exploited to specifically target single-nucleotide heterozygous mutations while exerting no aberrant effects on the wild-type alleles. Specifically, we target the heterozygous G13A activating mutation of KRAS in colorectal cancer cells and we show reversal of drug resistance to a MEK small-molecule inhibitor. Our study introduces a new paradigm in genome editing and therapeutic targeting via the use of gRNA to guide Cas9 to a desired protospacer adjacent motif.

## Introduction

**Fig 1:**
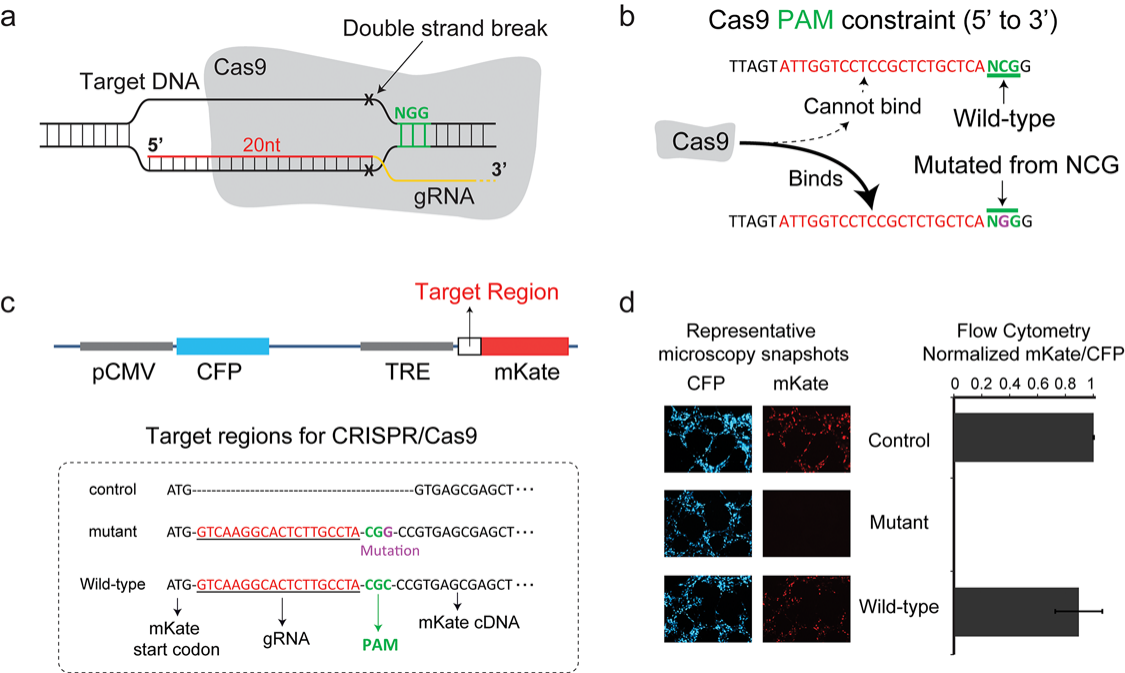
Protospacer Adjacent Mutated Motif-mediated targeting. (a) The gRNA-Cas9 complex targets DNA through complementarity with the gRNA sequence. Red color is the DNA binding domain. Green color is the PAM. (b) Example of the PAM constraint function for a random target sequence. (c) Schematic illustration of the fluorescence reporter plasmid. The variable regions, which correspond to either KRAS wild-type or p.G13A mutant alleles, were shown in the box and cloned after the mKate start codon. **(d)** Flow cytometry and fluorescence microcopy results for the normalized mKate expression. Both assays demonstrated that CRISPR specifically silenced the mKate carrying the KRAS p.G13A mutant sequence.

The Clustered Regularly Interspaced Short Palindromic Repeats (CRISPR) system, an immune system analog found in archaea and prokaryotes, allows a single guide RNA to direct a protein with combined helicase and nuclease activity to the DNA. CRISPR has revolutionized our ability to probe and edit the genome [1–7]. The CRISPR/Cas9 function relies on two fundamental components: (a) a guide RNA (gRNA) and (b) an endonuclease, such as the CRISPR associated (Cas) nuclease Cas9 derived from *Streptococcus pyogenes*. As illustrated in **Fig 1A**, upon base-pairing between the gRNA sequence and the target sequence in the genomic DNA, the Cas9-gRNA complex induces double strand break (DSB).

An often de-emphasized property of the CRISPR mechanism is that the Cas9 protein has a highly specific DNA binding constraint. In order for Cas9 to bind to DNA, the target sequence in the genomic DNA must be immediately followed by the correct Protospacer Adjacent Motif (PAM) sequence: 5’-NGG-3’ (**Fig 1A,** highlighted in green). While the Cas9-gRNA does allow for mismatches in the target sequence for a given gRNA [8], the vast majority of mutations in the PAM will fully abolish its binding and thereby desired function [9, 10]. We argue that this seemingly restrictive, binary binding property of Cas9 may facilitate a transformative approach in genome editing and targeting applications. Specifically, it points to the intriguing option of utilizing the gRNA in order to guide Cas9 to a desired protospacer adjacent mutated motif; in other words, a PAM sequence that has been formed as a result of a mutation in a wild-type allele (**Fig 1B**).

Here we demonstrate that we can exploit the PAM constraint in order to target and disrupt heterozygous single-nucleotide mutations in certain autosomal dominant (AD) disorders, while leaving the wild-type allele intact (**Fig 1B** and **S1 Fig**). As a proof of concept, we focus on single-nucleotide mutations in the human KRAS gene [11], which were demonstrated to be the underlying mechanism for acquired drug resistance to the MEK signaling inhibitor AZD6244 [12]. More specifically, the p.G13A (13^th^ codon of KRAS gene mutated from GGC to GCC) mutation generates a PAM sequence (5’-CGG-3’) on the antisense strand, which enables the efficient targeting of the corresponding Cas9-gRNA complex (the CRISPR target sequence: 5’-CGTCAAGGCACTCTTGCCTA-3’).

## Results

To confirm the hypothesis that we can exploit the Cas9 PAM DNA binding constraint, we first engineered plasmids that carry targets that emulate the KRAS p.G13A mutants (**S2 Fig and S1 Text**). More specifically, as illustrated in **Fig 1C**, we engineered plasmids that harbor a constitutive promoter (CMV) that controls CFP and an inducible promoter (TRE) that controls mKate. After the mKate start codon, in a variable target region, we introduced the p.G13A mutant and wild-type sequence. HEK293 cells with a stably integrated rtTA gene (it activates the TRE promoter in the presence of doxycycline), were transfected with 500ng of a plasmid that produces Cas9, 500ng of plasmid that produces the gRNA, and 100ng of either plasmid with the target regions. 24 hours later 1 μg/mL of doxycycline was added, and 48 hours post transfection cells were harvested for flow cytometry and fluorescence microscopy (**Fig 1D**). The results show that CRISPR completely silenced the mKate carrying the mutant sequence while the wild-type remained virtually unaffected.

We next explored whether the protospacer adjacent mutated motif -targeting approach can be adopted against endogenous genes. In particular we focused on KRAS p.G13A mutations. We first used CRISPR-based genome editing [13, 14] to generate a KRAS p.G13A mutant colorectal adenocarcinoma (SW48) stable cell line. Briefly, for the donor plasmid (**S3 Fig and S1 Text**), the left arm (~900bp) covers the DNA sequence immediately upstream of the expected cleavage region plus the first 10nts of the CRISPR target site (5’-AGTTATCTGAAATGTACCTT-3’), while the right arm (~900bp) covers the sequence downstream of the cleavage region plus the next 10nts of the target site. The target sequence was divided to avoid the cleavage of the donor plasmid during transfection. The left arm includes exon 2 of the KRAS gene, where the 13^th^ codon is located, and a GGC to GCC point mutation was introduced at that location in the donor plasmid using site-directed mutagenesis. Between the two arms, a FRT site-flanked puromycin resistance gene cassette was inserted. Upon Cas9-gRNA treatment, the p.G13A missense mutation was introduced through the homologous recombination mechanism under 2 μg/mL puromycin selection. To remove the puromycin resistance gene cassette, the positive clones were further transfected with plasmids expressing the hygromycin resistance gene and the FLP recombinase, followed by the selection of hygromycin B (150 μg/mL for 3 days). Subsequently, monoclonal stable cell lines were established using the limiting dilution method. We note that upon the removal of the puromycin resistance gene cassette, a “scar” sequence (the FRT site) of approximately 170 bp was left within the intron 3 of the KRAS gene.

**Fig 2:**
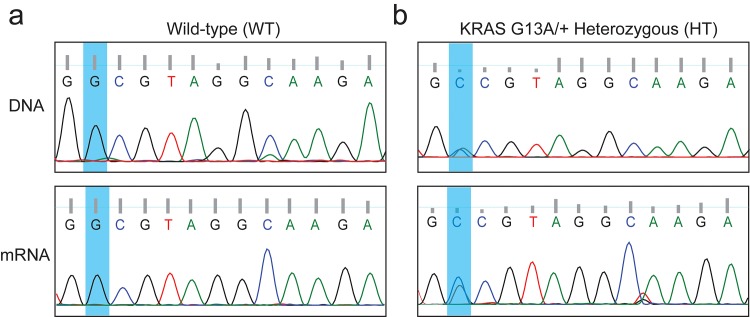
CRISPR/Cas9-mediated generation of a heterozygous KRAS G13A/+ stable SW48 cell line. Genomic DNA or mRNA were prepared from the KRAS wild type (WT) or G13A/+ SW48 (HT) cells and were used as PCR templates to isolate the cDNA fragments harboring the 13^th^ codon of the KRAS gene. **(a)** The Sanger sequencing results of the genomic DNA or mRNA samples from SW48 WT cells. **(b)** The Sanger sequencing results of the genomic DNA or mRNA samples from SW48 HT cells.

The genomic DNA or mRNA were then prepared from a monoclonal (modified to G13A) SW48 cell line, and subsequently were used as the PCR or RT-PCR templates to isolate the cDNAs harboring the 13^th^ codon of the KRAS gene. More specifically, primers P1 and P2 were used to amplify the genomic DNA fragment, and the 906 bp product was sequenced using P3 (**S1 Table**). Similarly, primers P4 and P5 were used to amplify the RT-PCR cDNA fragment, and the 222 bp product was sequenced using P2. As shown in **Fig 2B** (extended sequencing results at **S4–S7 Figs**), Sanger sequencing returned overlapping peaks of G and C at nucleotide position 38 (the nucleotide A of the ATG start codon of human KRAS gene was designated as position 1), indicating that it is a heterozygous clone (thereafter named as HT). In comparison, only the G peaks were observed in the wild-type (named as WT) samples (**Fig 2A**, and **S4–S7 Figs** for extended sequencing results).

We subsequently performed the drug sensitivity assay and observed that compared to its parental wild-type SW48 cells, the G13A stable cell line has increased resistance to the MEK inhibitor AZD6244 (**S8 Fig**). Specifically, under the treatment of 5 μM AZD6244, the relative cell viability (%) for WT cells were 35.9±1.1, while for HT cells 58.8±1.3.

We then introduced the Cas9 and G13A-targeting gRNA transcripts (**S2 Fig and S1 Text**) into the WT and HT SW48 cells using lentiviral delivery. Specifically, we used the lentiCRISPRv2 vector, which co-expresses a mammalian codon-optimized Cas9 nuclease along with a gRNA transcript [15]. 48 hours after transduction, the cells were selected using 2 μg/mL puromycin for 2 weeks. The resulting polyclonal SW48 cells were named as WT-Cas9 and HT-Cas9 cells, respectively.

**Fig 3:**
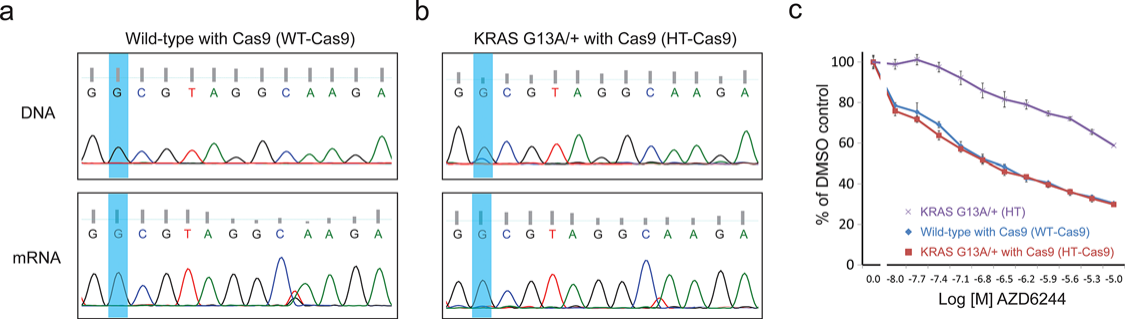
Protospacer Adjacent Mutated Motif-mediated targeting of the KRAS p.G13A mutation in colorectal cancer cells. Genomic DNA or mRNA were prepared from the KRAS WT-Cas9 or HT-Cas9 cells and were used as PCR templates to isolate the cDNA fragments harboring the 13^th^ codon of the KRAS gene. **(a)** The Sanger sequencing results of the genomic DNA or mRNA samples from SW48 WT-Cas9 cells. **(b)** The Sanger sequencing results of the genomic DNA or mRNA samples from SW48 HT-Cas9 cells. **(c)** The drug response curves of the KRAS HT, WT-Cas9 and HT-Cas9 SW48 cells to the MEK inhibitor AZD6244. The WT-Cas9 and HT-Cas9 cell lines show the same AZD6244 sensitivity.

Subsequently, the genomic DNA or mRNA were again prepared and used as the PCR or RT-PCR templates to determine the status at the 13^th^ codon. As shown in **Fig 3A** (extended sequencing in S9–S12 **Figs**) and **Fig 2A**, the genomic DNA and mRNA profiles between WT and WT-Cas9 cells were the same, indicating no detectable editing events on the KRAS wild-type allele by the G13A-targeting CRISPR complex. In contrast, while the heterozygous cells have overlapping peaks of G and C at the 13^th^ codon, the HT-Cas9 cells returned strictly G signals (**Fig 2B** and **Fig 3B**).

These results confirm our hypothesis that the CRISPR complex would specifically target the G13A mutant allele. Furthermore, in the HT-Cas9 cells we observe increased noise signal in the genomic DNA profile which is presumably due to the non-homologous end joining (NHEJ) events. These include insertions at the DNA double-strand breaks or deletions after the 13^th^ codon (**S11 Fig**). Importantly, these edits do not yield functional mRNAs (**Fig 3B**).

We subjected the two polyclonal cell lines (WT-Cas9 and HT-Cas9) to the AZD6244 sensitivity assay. We observe that the Cas9-treated wild-type and heterozygous cells have similar drug sensitivity (**Fig 3C)**, thus a reversal in the MEK resistance due to the gain-of-function KRAS mutation (**S8 Fig**). Specifically, under the treatment of 5μM AZD6244, the relative cell viability (%) for WT-Cas9 cells were 30.4±0.2, while for HT-Cas9 cells 29.8±1.7, indicating that the increased resistance to the MEK inhibitor AZD6244 of the heterozygous cells is corrected by specifically targeting the activating G13A mutation. We also observed that compared to the wild-type SW48 cells, the Cas9 expression mildly reduced the overall cell viability (**S8 Fig**).

Here we demonstrated that the CRISPR/Cas9 system can efficiently target gain-of-function point mutations by exploiting the PAM constraint. We note that an alternative genome editing tool, the Transcription Activator-Like Effector Nucleases (TALENs) [16–18], has a comparable DNA binding constraint. More specifically, the 5’-most base of the DNA target sequence bound by the TALEN (termed as the N0 base) should be a thymine (T) [19], which renders the technology suitable for a wide range of target mutations. We show as an example (**S13 and S14 Figs**), that the p.D12G mutation of human KRAS gene results in a T nucleotide in the mutant allele, which can be targeted efficiently by engineered TALENs.

## Discussion

Constraining the CRISPR/Cas activity to a single allele was described recently using a single-nucleotide polymorphism (SNP) difference within the target sequence [20]. Here, we demonstrate an alternative approach in genome editing and therapeutic targeting via the use of gRNA to guide the Cas9 to desired protospacer adjacent motifs. The advantage of targeting the PAM sequence is that it may enhance the single-allelic targeting specificity. For its use in therapeutic applications, there are several autosomal dominant somatic mutations that can be targeted. We emphasize that such mutations have been detected in a broad range of disorders beyond cancer. As an example, the CRISPR/Cas9 complex was recently shown to efficiently target both *in vitro* and *in vivo KRT12* mutant allele containing a PAM motif [21].

A current limitation of our approach is that the candidate mutations should result in the specific Cas9 PAM sequence (5’-NGG-3’). Nevertheless, considering the diversity of Cas proteins [22–24] and the active protein engineering efforts to modify them [25, 26] we expect that a broad range of PAM options will be available before long. Recently, *Streptococcus pyogenes* Cas9 derivatives were shown to recognize alternative PAM sequences with comparable editing efficiency and more stringent PAM-binding specificities than their wild-type counterpart [27].

## Materials and Methods

### Cell culture and transient transfection

The HEK293 cells were acquired from the American Type Culture Collection (ATCC, catalog number: CRL-1573). The wild type SW48 cells were purchased from Horizon Discovery (catalog number: HD 103–011). Both cell lines were maintained at 37°C, 100% humidity and 5% CO_2_. The cells were grown in Dulbecco’s modified Eagle’s medium (DMEM, Invitrogen, catalog number: 11965–1181) supplemented with 10% Fetal Bovine Serum (FBS, Invitrogen, catalog number: 26140), 0.1 mM MEM non-essential amino acids (Invitrogen, catalog number: 11140–050), and 0.045 units/mL of Penicillin and 0.045 units/mL of Streptomycin (Penicillin-Streptomycin liquid, Invitrogen, catalog number: 15140). To pass the cells, the adherent culture was first washed with PBS (Dulbecco’s Phosphate Buffered Saline, Mediatech, catalog number: 21-030-CM), then trypsinized with Trypsin-EDTA (0.25% Trypsin with EDTAX4Na, Invitrogen, catalog number: 25200) and finally diluted in fresh medium.

For transient transfection, ~300,000 cells in 1 mL of complete medium were plated into each well of 12-well culture treated plastic plates (Griener Bio-One, catalog number: 665180) and grown for 16–20 hours. All transfections were then performed using 1.75 μL of JetPRIME (Polyplus Transfection) and 75 μL of JetPRIME buffer. The transfection mixture was then applied to the cells and mixed with the medium by gentle shaking. When applicable, doxycycline (Clontech, catalog number: 631311) was added after transfection.

### Fluorescence microscopy

Microscopy was performed 48–72 hours post transfection. The live cells were grown on 12-well plates (Greiner Bio-One) in the complete medium. Cells were imaged using an Olympus IX81 microscope in a Precision Control environmental chamber. The images were captured using a Hamamatsu ORCA-03 Cooled monochrome digital camera. The filter sets (Chroma) are as follows: ET436/20x (excitation) and ET480/40 m (emission) for CFP, ET560/40x (excitation) and ET630/75 m (emission) for mKate. Data collection and processing was performed in the software package Slidebook 5.0. All images within a given experimental set were collected with the same exposure times and underwent identical processing.

### Flow cytometry

48–72 hours post transfection cells from each well of the 12-well plates were trypsinized with 0.1 mL 0.25% Trypsin-EDTA at 37°C for 3 min. Trypsin-EDTA was then neutralized by adding 0.9 mL of complete medium. The cell suspension was centrifuged at 1,000 rpm for 5 min and after removal of supernatants, the cell pellets were re-suspended in 0.5 mL PBS buffer. The cells were analyzed on a BD LSRFortessa flow analyzer. CFP was measured with a 445-nm laser and a 515/20 band-pass filter, and mKate with a 561-nm laser, 610 emission filter and 610/20 band-pass filter. For data analysis, 100,000 events were collected. A FSC (forward scatter)/SSC (side scatter) gate was generated using a un-transfected negative sample and applied to all cell samples. The mKate and CFP readings from un-transfected HEK293 cells were set as baseline values and were subtracted from all other experimental samples. The normalized mKate values (mKate/CFP) were then collected and processed by FlowJo. All experiments were performed in triplicates.

### Generation of SW48 G13A/+ monoclonal stable cell line

To generate the G13A/+ monoclonal stable cell lines, ~10 million of the human SW48 cells were seeded onto a 10 cm petri dish. 16 hours later, the cells were transiently transfected with 3.3 μg of the donor plasmid, 3.3 μg of the U6-gRNA construct, and 3.3 μg of the PCMV-Cas9 plasmid using the JetPRIME reagent (Polyplus Transfection). 48 hours later, puromycin (Life Technologies, catalog number: A1113803) was added at the final concentration of 2 μg/mL. The selection lasted ~2 weeks, after which the surviving clones were pooled to generate the polyclonal stable cells. To remove the puromycin resistance gene cassette, ~10 million of the polyclonal cells were seeded onto a 10 cm petri dish, and after 16 hours were transfected with 5 μg of hygromycin resistance gene plasmid (unpublished data) and 5 μg of Flpase (unpublished data) using the JetPRIME reagent. 48 hours later, the cells were treated with hygromycin B (150 μg/mL, Life Technologies) for 3 days. The surviving cells were pooled to generate the polyclonal stable cells, from which monoclonal stable cells were isolated using the limiting dilution method. Specifically, the isolated clones were selected using cloning cylinders (Corning, catalog number: 22877–256) and further expanded and maintained in the complete growth medium containing 2 μg/mL of puromycin.

### Profiling the KRAS G13A mutations in SW48 cells

To profile the KRAS G13A mutations using the genomic DNA samples, the total genomic DNA was isolated from SW48 cells using the DNeasy Blood&Tissue Kit (Qiagen). Subsequently, the cDNA fragments harboring the 13^th^ codon of the KRAS gene were PCR amplified by using ~50 ng of the genomic DNA and primers P1 and P2. The PCR conditions were: first one cycle of 30 s at 95°C, followed by 30 cycles of 10 s at 95°C, 30 s at 58°C, and 1 min at 72°C. The 906 bp product was then subjected to direct Sanger sequencing using primer P3 and analyzed using FinchTV (Geospiza). To profile the KRAS G13A mutations using the mRNA samples, total RNAs were extracted from SW48 cells using the RNeasy Mini Kit (Qiagen). First-strand cDNA synthesis was then performed using 500 ng of the RNA sample using QuantiTect Reverse Transcription Kit (Qiagen) in a 10 μL reaction. The reaction mixture was then diluted 100X using dH_2_0, and 1 μL of the diluent was used as the template for PCR reactions together with primers P4 and P5 to isolate the cDNA fragments harboring the 13^th^ codon of the KRAS gene. The PCR conditions were: first one cycle of 30 s at 95°C, followed by 35 cycles of 10 s at 95°C, 30 s at 58°C, and 30 s at 72°C. The 222 bp product was then subjected to direct Sanger sequencing using primer P5 and analyzed using FinchTV (Geospiza).

### Cell viability assay

Approximately 4000 of the SW48 cells were seeded into 96-well plates in 100 μL of the complete medium. 16 hours later, the cells were treated with MEK inhibitor (AZD6244) at various concentrations. 96 hours post drug treatment, the cell viability assays were performed using the CellTiter-Glo Luminescent Cell Viability Assay kit (Promega, catalog number: G7570) according to the manufacturer’s protocol. All experiments were performed in 4 replicates.

### Generation of the KRAS G13A-targeting lentiviral vectors

The LentiCRISPRv2 system was purchased from Addgene (catalog number: 52961) (15). The LentiCRISPRv2-gRNA construct was first prepared using primers P25 and P26 following the vendor’s protocol. The gRNA sequence was confirmed by Sanger sequencing using primer P27. To generate the lentiviral vectors, the HEK293T cells were seeded at 50–70% before transfection and then transfected with 3.3 μg of the LentiCRISPRv2-gRNA plasmid, 3.3 μg of the pMD2-VSVG plasmid, and 3.3 μg of the psPAX2 plasmid using JetPRIME. 24 hours later, the medium were removed and replenished with 5 mL of the complete growth medium. The lentiviral stocks were then harvested in the next three days and the pooled 15 mL growth medium were centrifuged at 3,000 rpm for 15 min at 4°C to remove the cell debris. The supernatant were filtered through 0.45 μm filter, dispensed into 1–2 mL aliquots and stored at -80°C. To generate the SW48 WT-CRISPR and HT-CRISPR stable cells, ~10 million cells were seeded onto a 10-cm petri dish. 16 hours later, the cells were transduced using 1 mL of the lentiviral vectors. 48 hours post transduction, the cells were treated with 2 μg/mL of puromycin, and the polyclonal stable cell lines were established after ~2 weeks of drug selection.

## Supporting Information

S1 Fig Schematic illustration of the PAM constraint-mediated genome editing by the CRISPR complex. (DOCX)

S2 Fig Specific location of KRAS mutants (p.G13A) and associated CRISPR target. (DOCX)

S3 Fig Nucleotide sequence of the donor plasmid for generation of the G13A/+ SW48 cells. (DOCX)

S4 Fig The Sanger sequencing results of the genomic DNA from SW48 WT cells. (DOCX)

S5 Fig The Sanger sequencing results of the mRNA from SW48 WT cells. (DOCX)

S6 Fig The Sanger sequencing results of the genomic DNA from SW48 HT cells. (DOCX)

S7 Fig The Sanger sequencing results of the mRNA from SW48 HT cells. (DOCX)

S8 Fig The KRAS G13A/+ stable SW48 cell line demonstrated increased resistance to AZD6244. (DOCX)

S9 Fig The Sanger sequencing results of the genomic DNA from SW48 WT-Cas9 cells. (DOCX)

S10 Fig The Sanger sequencing results of the mRNA from SW48 WT-Cas9 cells. (DOCX)

S11 Fig The Sanger sequencing results of the genomic DNA from SW48 HT-Cas9 cells. (DOCX)

S12 Fig The Sanger sequencing results of the mRNA from SW48 HT-Cas9 cells. (DOCX)

S13 Fig Schematic illustration of the reporter plasmid for exploring the N0 TALEN constraint. (DOCX)

S14 Fig TALEN N0 constraint-mediated genome editing of the KRAS p.D12G mutations *in vitro*. (DOCX)

S1 Table Primers used in this study. (DOCX)

S1 Text General cloning protocols and DNA constructs. (DOCX)
