# Supplementary material for: Exploiting the CRISPR/Cas9 PAM Constraint for Single-Nucleotide Resolution Interventions
Source: PLoS One. 2016 Jan 20;11(1):e0144970. doi: 10.1371/journal.pone.0144970 (PMC4720446; doi:10.1371/journal.pone.0144970)
Supplement: S1 Fig — (DOCX) [file pone.0144970.s001.docx]

**Figure S1**


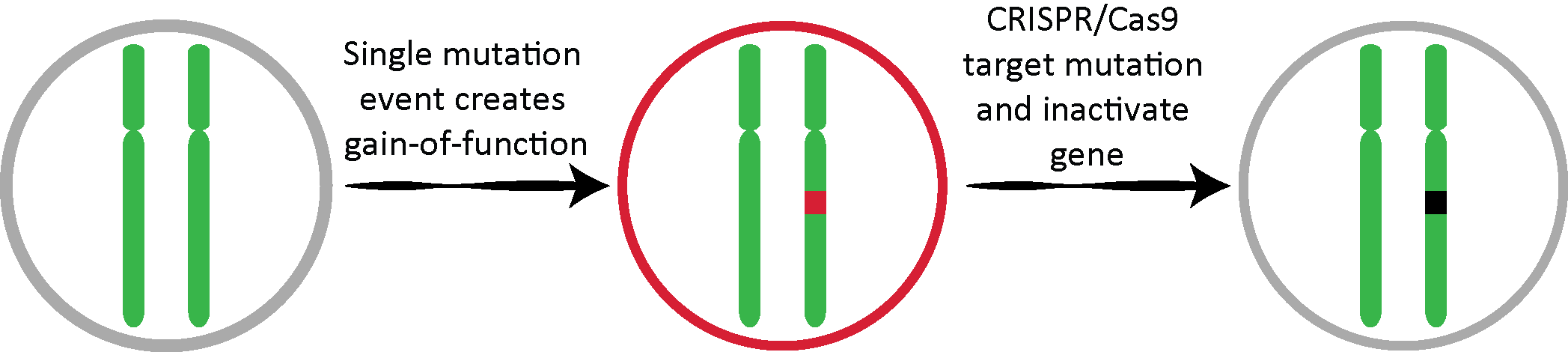


S1 Fig: Schematic illustration of the PAM constraint-mediated genome editing by the CRISPR complex.
