# Supplementary material for: Exploiting the CRISPR/Cas9 PAM Constraint for Single-Nucleotide Resolution Interventions
Source: PLoS One. 2016 Jan 20;11(1):e0144970. doi: 10.1371/journal.pone.0144970 (PMC4720446; doi:10.1371/journal.pone.0144970)
Supplement: S2 Fig — (DOCX) [file pone.0144970.s002.docx]

**Figure S2**


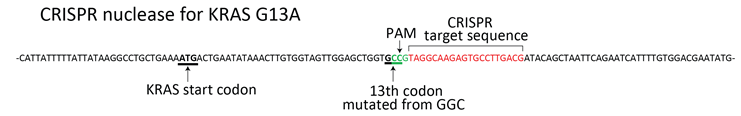


**S2 Fig: Specific location of KRAS mutants (p.G13A) and associated CRISPR target.** The red color is the DNA binding domain, the green is PAM, and the underlined are specific codons. Note that the CRISPR binds on the antisense strand.
