# Supplementary material for: Exploiting the CRISPR/Cas9 PAM Constraint for Single-Nucleotide Resolution Interventions
Source: PLoS One. 2016 Jan 20;11(1):e0144970. doi: 10.1371/journal.pone.0144970 (PMC4720446; doi:10.1371/journal.pone.0144970)
Supplement: S3 Fig — (DOCX) [file pone.0144970.s003.docx]

**Figure S3**

gtcgacatcgatcacacattaaaatgtcgtcaaatattgttcttctttgcctcagtgtttaaatttttatttccccatgacacaatccagctttatttgacactcattctctcaactctcatctgattcttactgttaatatttatccaagagaactactgccatgatgctttaaaagtttttctgtagctgttgcatattgacttctaacacttagaggtgggggtccactaggaaaactgtaacaataagagtggagatagctgtcagcaacttttgtgagggtgtgctacagggtgtagagcactgtgaagtctctacatgagtgaagtcatgatatgatcctttgagagcctttagccgccgcagaacagcagtctggctatttagatagaacaacttgattttaagataaaagaactgtctatgtagcatttatgcatttttcttaagcgtcgatggaggagtttgtaaatgaagtacagttcattacgatacacgtctgcagtcaactggaattttcatgattgaattttgtaaggtattttgaaataatttttcatataaaggtgagtttgtattaaaaggtactggtggagtatttgatagtgtattaaccttatgtgtgacatgttctaatatagtcacattttcattatttttattataaggcctgctgaaaatgactgaatataaacttgtggtagttggagctggt**GCC**gtaggcaagagtgccttgacgatacagctaattcagaatcattttgtggacgaatatgatccaacaatagaggtaaatcttgttttaatatgcatattactggtgcaggaccattctttgatacagataaaggtttctctgaccattttcatgagtacttattacaagataattatgctgaaagttaagttatctgagagctcctgggacggaggcttgtttgcgaggccgcggccggccgaagttcctattctctagaaagtataggaacttctaccgggtaggggaggcgcttttcccaaggcagtctggagcatgcgctttagcagccccgctgggcacttggcgctacacaagtggcctctggcctcgcacacattccacatccaccggtaggcgccaaccggctccgttctttggtggccccttcgcgccaccttctactcctcccctagtcaggaagttcccccccgccccgcagctcgcgtcgtgcaggacgtgacaaatggaagtagcacgtctcactagtctcgtgcagatggacagcaccgctgagcaatggaagcgggtaggcctttggggcagcggccaatagcagctttgctccttcgctttctgggctcagaggctgggaaggggtgggtccgggggcgggctcaggggcgggctcaggggcggggcgggcgcccgaaggtcctccggaggcccggcattctgcacgcttcaaaagcgcacgtctgccgcgctgttctcctcttcctcatctccgggcctttcgacctgcatccatctagatctcgatcgagcagctgaagcttaccatgaccgagtacaagcccacggtgcgcctcgccacccgcgacgacgtccccagggccgtacgcaccctcgccgccgcgttcgccgactaccccgccacgcgccacaccgtcgatccggaccgccacatcgagcgggtcaccgagctgcaagaactcttcctcacgcgcgtcgggctcgacatcggcaaggtgtgggtcgcggacgacggcgccgcggtggcggtctggaccacgccggagagcgtcgaagcgggggcggtgttcgccgagatcggcccgcgcatggccgagttgagcggttcccggctggccgcgcagcaacagatggaaggcctcctggcgccgcaccggcccaaggagcccgcgtggttcctggccaccgtcggcgtctcgcccgaccaccagggcaagggtctgggcagcgccgtcgtgctccccggagtggaggcggccgagcgcgccggggtgcccgccttcctggagacctccgcgccccgcaacctccccttctacgagcggctcggcttcaccgtcaccgccgacgtcgaggtgcccgaaggaccgcgcacctggtgcatgacccgcaagcccggtgcctgagggaggctaactgaagcttcccgggggtaccaaattcgtcgacagatctaacttgtttattgcagcttataatggttacaaataaagcaatagcatcacaaatttcacaaataaagcatttttttcactgcattctagttgtggtttgtccaaactcatcaatgtatcttatgatgtctgcatatggaagttcctattctctagaaagtataggaacttcgcggccgctcccacccgctcgtccccccgcgcacctttgctaggagcgggtcgcccgagctcaatgtaccttgggtttcaagttatatgtaaccattaatatgggaactttactttccttgggagtatgtcagggtccatgatgttcactctctgtgcattttgattggaagtgtatttcagagtttcgtgagagggtagaaatttgtatcctatctggacctaaaagacaatctttttattgtaacttttatttttatgggtttcttggtattgtgacatcatatgtaaaggttagatttaattgtactagtgaaatataattgtttgatggttgatttttttaaacttcatcagcagtattttcctatcttcttctcaacattagagaacctacaactaccggataaattttacaaaatgaattatttgcctaaggtgtggtttatataaaggtactattaccaactttacctttgctttgttgtcatttttaaatttactcaaggaaatactaggatttaaaaaaaaattccttgagtaaatttaaattgttatcatgtttttgaggattattttcagatttttttagtttaatgaaaatttaccaaagtaaagaccagcagcagaatgataagtaaagacctgtaagacaccttgaaggtcatggagtagaacttccatcccaagcagatgaggatttatttaatctcaaagacctccaggaggggacattccccaactgtccttgttaactcattttcagaacatatttattagcatattttacatgtaatttggatcttcatgttaaatttaacatcagtggagatggaaaataagcatatcgccttgtctttgaaatagccctatattgttagattgtttcttaggcttctttaccctgggttaagcagtcctaatactttagcgaattctgcagtcgacggtacccggccgcgactctagatcataatcagCtcgagcttaacaagcttcgaaacgatatgggctgaatacaaaaacgatatgggctgaatacaaaaacgatatgggctgaatacaaaccgcttgaagtctttaattaaaccgcttgaagtctttaattaaaccgcttgaagtctttaattaaaggatccaccggatctagataactgatcataatcgcggccgcactcctcaggtgcaggctgcctatcagaaggtggtggctggtgtggccaatgccctggctcacaaataccactgagatctttttccctctgccaaaaattatggggacatcatgaagccccttgagcatctgacttctggctaataaaggaaatttattttcattgcaatagtgtgttggaattttttgtgtctctcactcggaaggacatatgggagggcaaatcatttaaaacatcagaatgagtatttggtttagagtttggcaacatatgccatatgctggctgccatgaacaaaggtggctataaagaggtcatcagtatatgaaacagccccctgctgtccattccttattccatagaaaagccttgacttgaggttagattttttttatattttgttttgtgttatttttttctttaacatccctaaaattttccttacatgttttactagccagatttttcctcctctcctgactactcccagtcatagctgtccctcttctcttatgaagatccctcgacctgcagcccaagcttggcgtaatcatggtcatagctgtttcctgtgtgaaattgttatccgctcacaattccacacaacatacgagccggaagcataaagtgtaaagcctggggtgcctaatgagtgagctaactcacattaattgcgttgcgctcactgcccgctttccagtcgggaaacctgtcgtgccagcggatccgcatctcaattagtcagcaaccatagtcccgcccctaactccgcccatcccgcccctaactccgcccagttccgcccattctccgccccatggctgactaattttttttatttatgcagaggccgaggccgcctcggcctctgagctattccagaagtagtgaggaggcttttttggaggcctaggcttttgcaaaaagctaacttgtttattgcagcttataatggttacaaataaagcaatagcatcacaaatttcacaaataaagcatttttttcactgcattctagttgtggtttgtccaaactcatcaatgtatcttatcatgtctggatccgctgcattaatgaatcggccaacgcgcggggagaggcggtttgcgtattgggcgctcttccgcttcctcgctcactgactcgctgcgctcggtcgttcggctgcggcgagcggtatcagctcactcaaaggcggtaatacggttatccacagaatcaggggataacgcaggaaagaacatgtgagcaaaaggccagcaaaaggccaggaaccgtaaaaaggccgcgttgctggcgtttttccataggctccgcccccctgacgagcatcacaaaaatcgacgctcaagtcagaggtggcgaaacccgacaggactataaagataccaggcgtttccccctggaagctccctcgtgcgctctcctgttccgaccctgccgcttaccggatacctgtccgcctttctcccttcgggaagcgtggcgctttctcaatgctcacgctgtaggtatctcagttcggtgtaggtcgttcgctccaagctgggctgtgtgcacgaaccccccgttcagcccgaccgctgcgccttatccggtaactatcgtcttgagtccaacccggtaagacacgacttatcgccactggcagcagccactggtaacaggattagcagagcgaggtatgtaggcggtgctacagagttcttgaagtggtggcctaactacggctacactagaaggacagtatttggtatctgcgctctgctgaagccagttaccttcggaaaaagagttggtagctcttgatccggcaaacaaaccaccgctggtagcggtggtttttttgtttgcaagcagcagattacgcgcagaaaaaaaggatctcaagaagatcctttgatcttttctacggggtctgacgctcagtggaacgaaaactcacgttaagggattttggtcatgagattatcaaaaaggatcttcacctagatccttttaaattaaaaatgaagttttaaatcaatctaaagtatatatgagtaaacttggtctgacagttaccaatgcttaatcagtgaggcacctatctcagcgatctgtctatttcgttcatccatagttgcctgactccccgtcgtgtagataactacgatacgggagggcttaccatctggccccagtgctgcaatgataccgcgagacccacgctcaccggctccagatttatcagcaataaaccagccagccggaagggccgagcgcagaagtggtcctgcaactttatccgcctccatccagtctattaattgttgccgggaagctagagtaagtagttcgccagttaatagtttgcgcaacgttgttgccattgctacaggcatcgtggtgtcacgctcgtcgtttggtatggcttcattcagctccggttcccaacgatcaaggcgagttacatgatcccccatgttgtgcaaaaaagcggttagctccttcggtcctccgatcgttgtcagaagtaagttggccgcagtgttatcactcatggttatggcagcactgcataattctcttactgtcatgccatccgtaagatgcttttctgtgactggtgagtactcaaccaagtcattctgagaatagtgtatgcggcgaccgagttgctcttgcccggcgtcaatacgggataataccgcgccacatagcagaactttaaaagtgctcatcattggaaaacgttcttcggggcgaaaactctcaaggatcttaccgctgttgagatccagttcgatgtaacccactcgtgcacccaactgatcttcagcatcttttactttcaccagcgtttctgggtgagcaaaaacaggaaggcaaaatgccgcaaaaaagggaataagggcgacacggaaatgttgaatactcatactcttcctttttcaatattattgaagcatttatcagggttattgtctcatgagcggatacatatttgaatgtatttagaaaaataaacaaataggggttccgcgcacatttccccgaaaagtgccacctg

**S3 Fig: Nucleotide sequence of the donor plasmid for generation of the G13A/+ SW48 cells.** The left arm was highlighted in yellow. The right arm was highlighted in purple. The puromycin resistance gene cassette was highlighted in grey. The two FRT sites were highlighted in red. The CRISPR target sites (20nts) were divided into two 10nts sequences and highlighted in blue. The KRAS 13^th^ codon, which was mutated from GGC to GCC, was in capital letters and underlined.
