# Supplementary figures and images for: Exploiting the CRISPR/Cas9 PAM Constraint for Single-Nucleotide Resolution Interventions
Source: PLoS One. 2016 Jan 20;11(1):e0144970. doi: 10.1371/journal.pone.0144970 (PMC4720446; doi:10.1371/journal.pone.0144970)

**Figure S4**


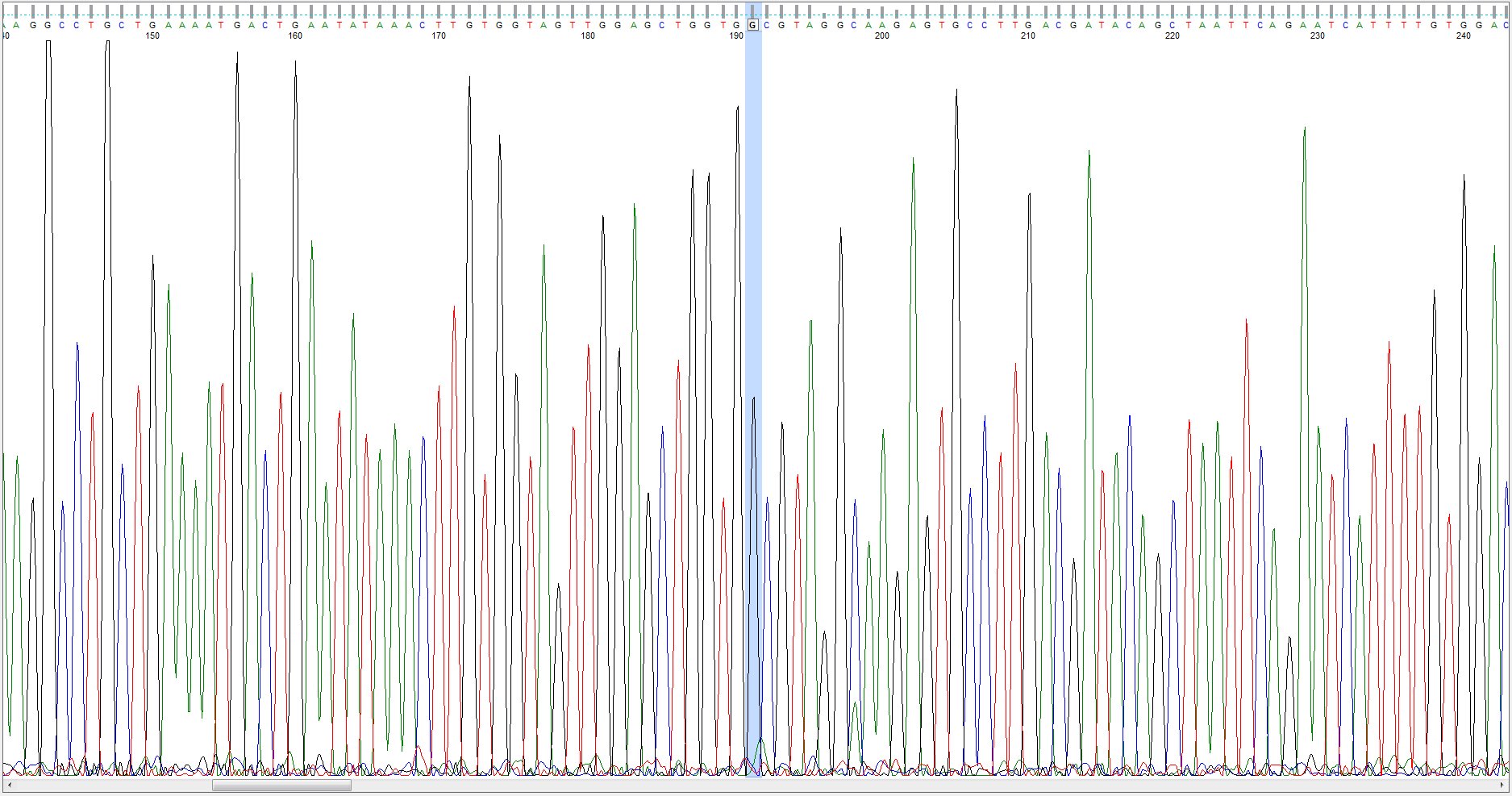


**S4 Fig. The Sanger sequencing results of the genomic DNA from SW48 WT cells.**

Supplement: S4 Fig — (DOCX) [file pone.0144970.s004.docx]

**Figure S5**


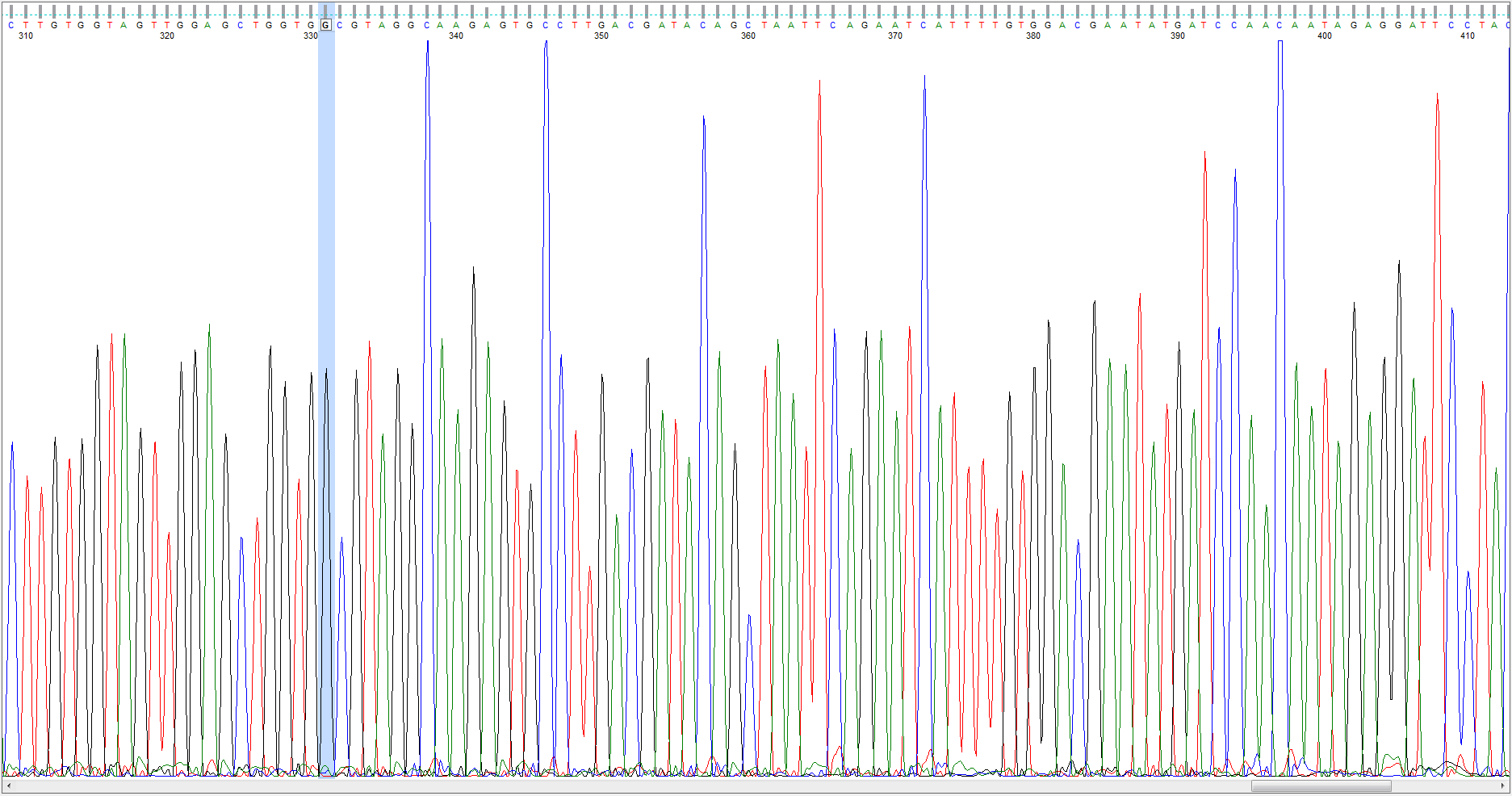


**S5 Fig. The Sanger sequencing results of the mRNA from SW48 WT cells.**

Supplement: S5 Fig — (DOCX) [file pone.0144970.s005.docx]

**Figure S6**


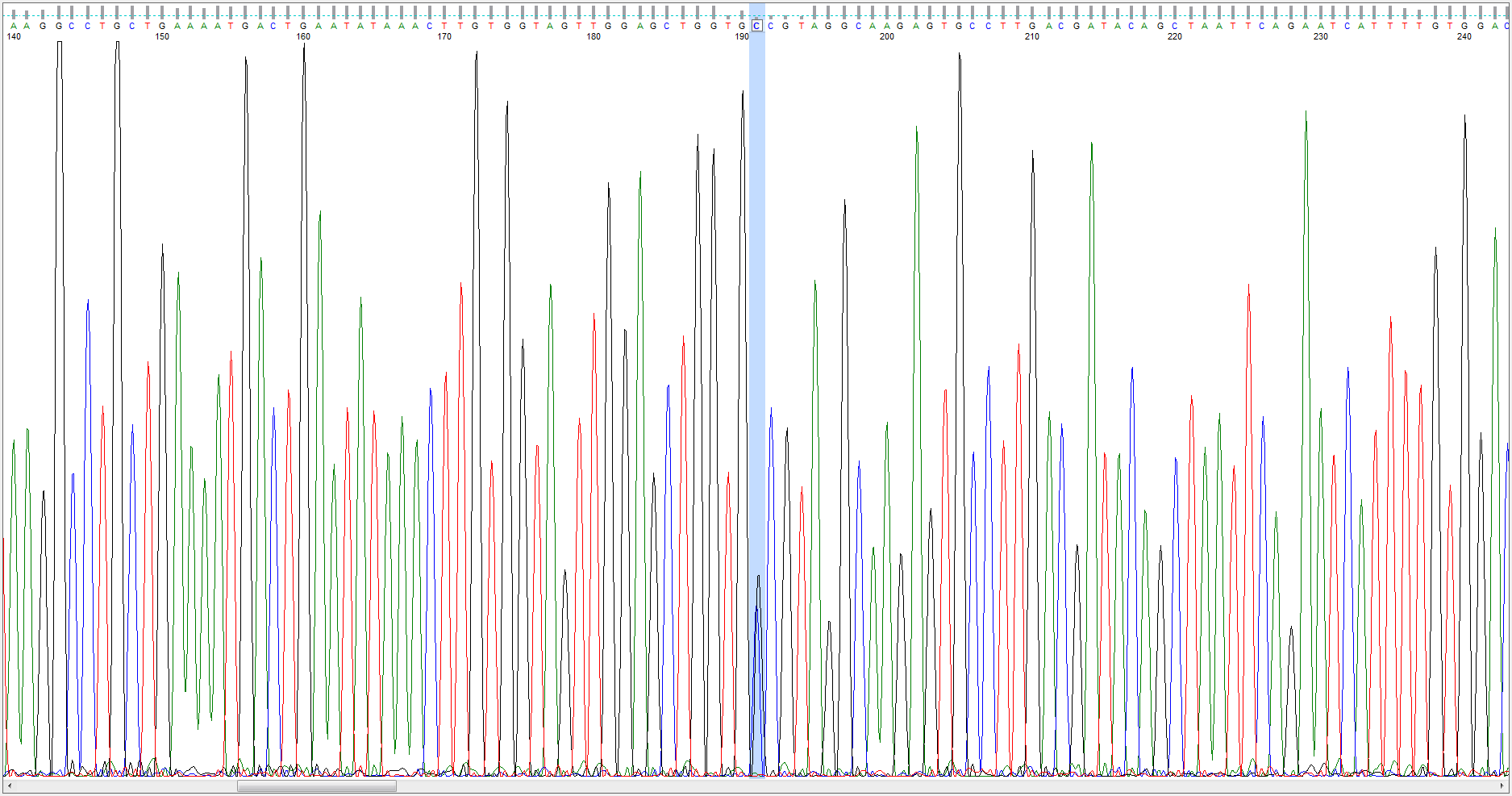


**S6 Fig. The Sanger sequencing results of the genomic DNA from SW48 HT cells.**

Supplement: S6 Fig — (DOCX) [file pone.0144970.s006.docx]

**Figure S7**


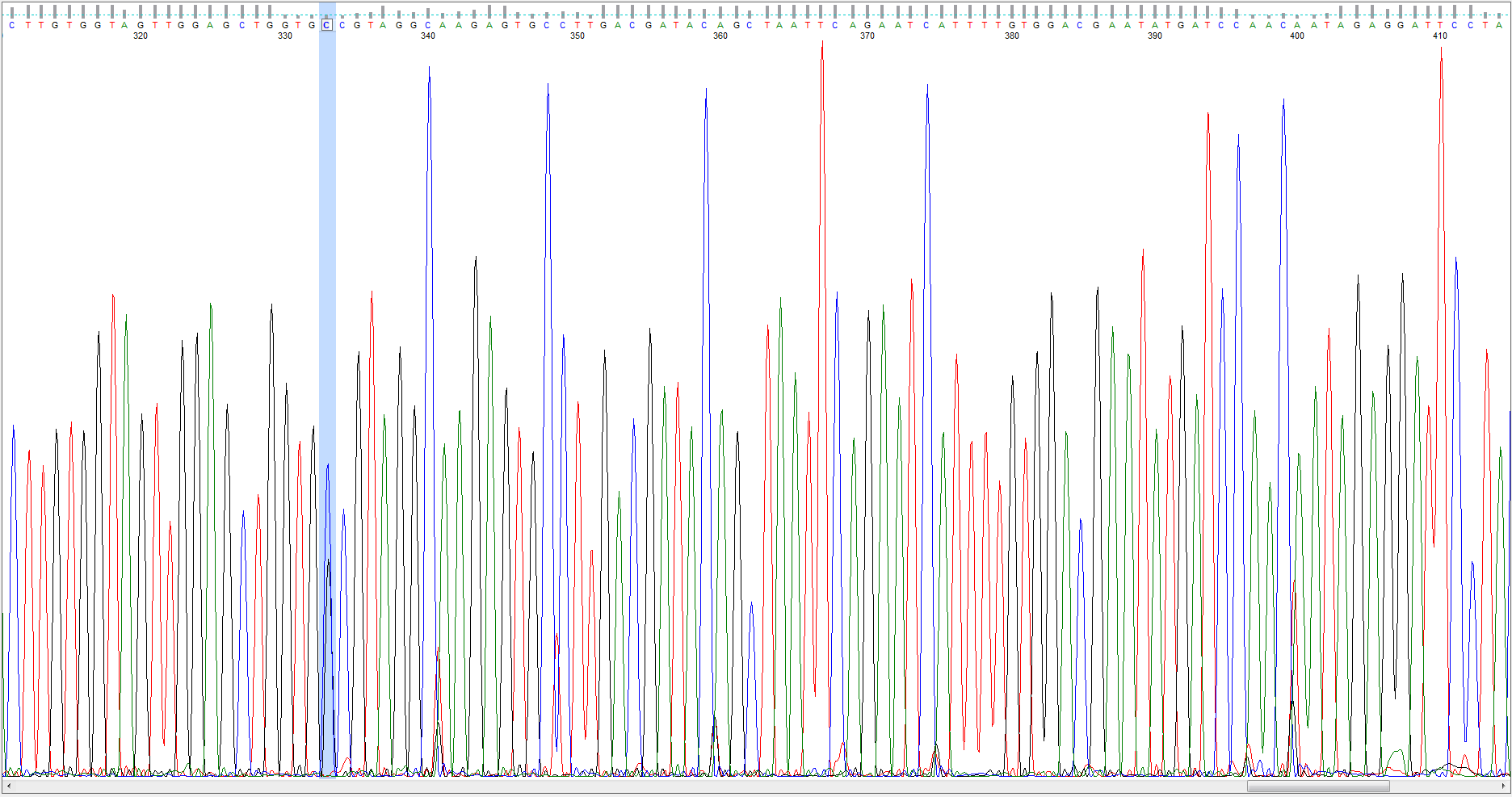


**S7 Fig. The Sanger sequencing results of the mRNA from SW48 HT cells.**

Supplement: S7 Fig — (DOCX) [file pone.0144970.s007.docx]

**Figure S9**


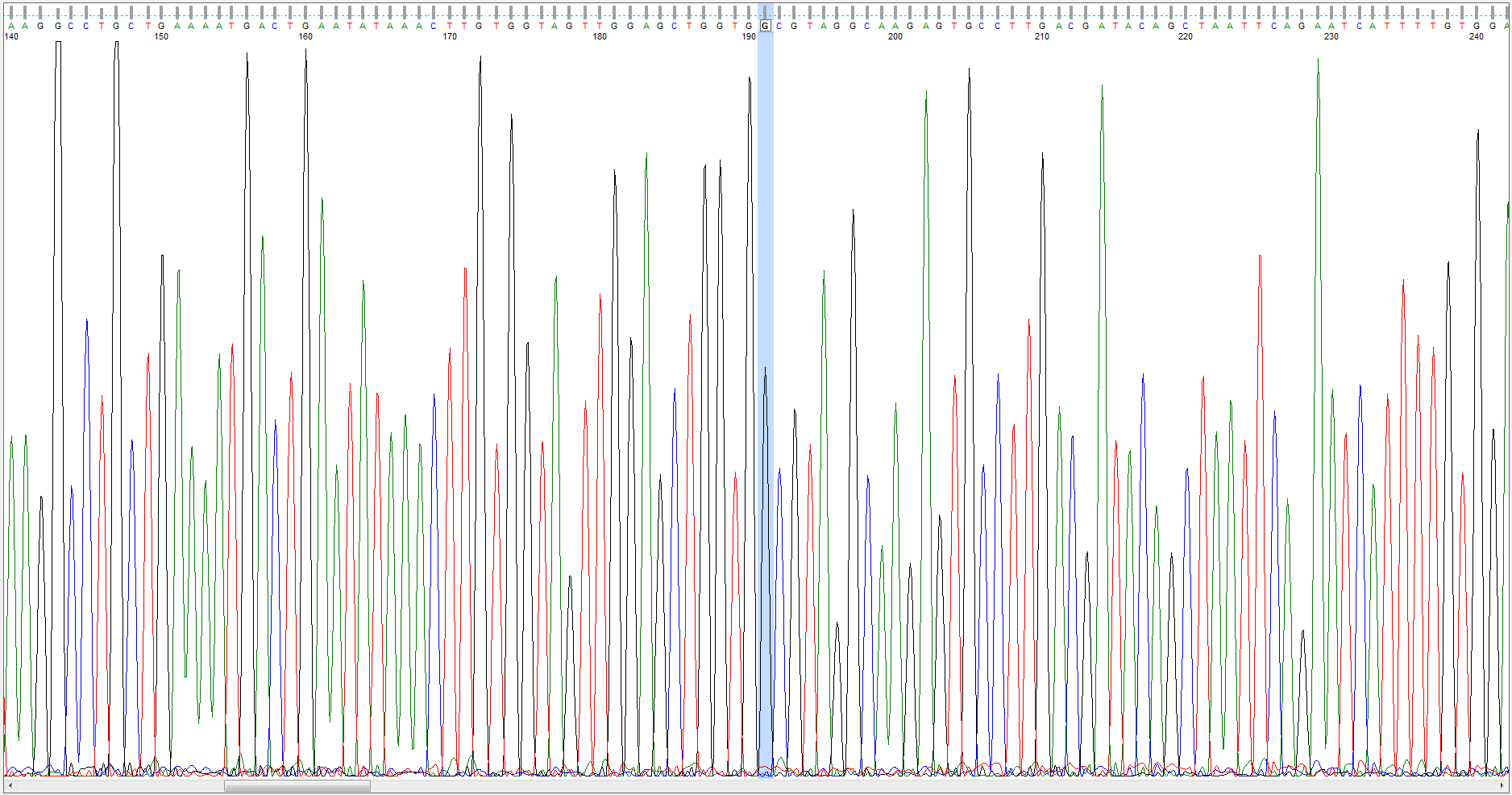


**S9 Fig. The Sanger sequencing results of the genomic DNA from SW48 WT-Cas9 cells.**

Supplement: S9 Fig — (DOCX) [file pone.0144970.s009.docx]

**Figure S10**


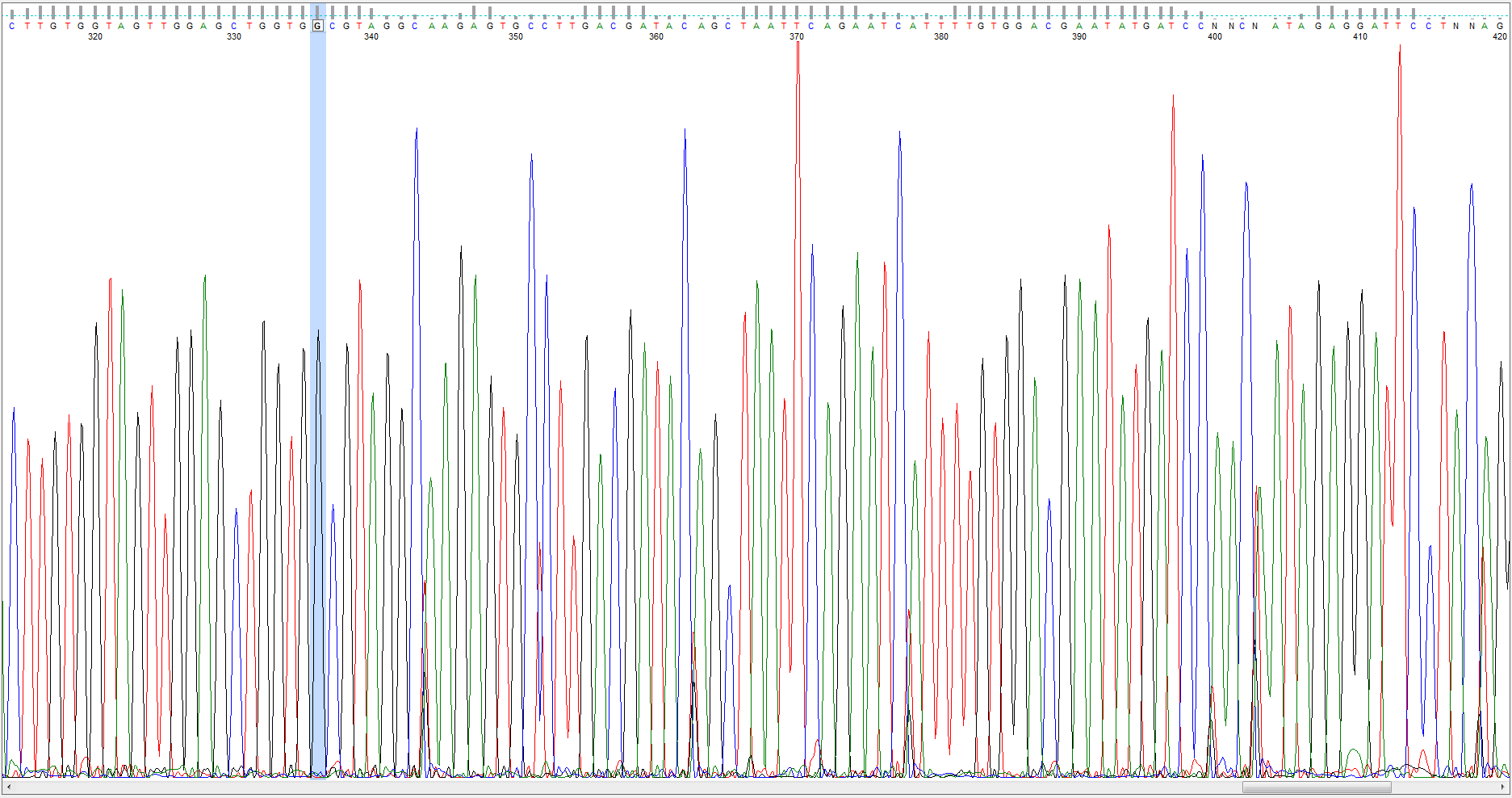


**S10 Fig. The Sanger sequencing results of the mRNA from SW48 WT-Cas9 cells.**

Supplement: S10 Fig — (DOCX) [file pone.0144970.s010.docx]

**Figure S11**


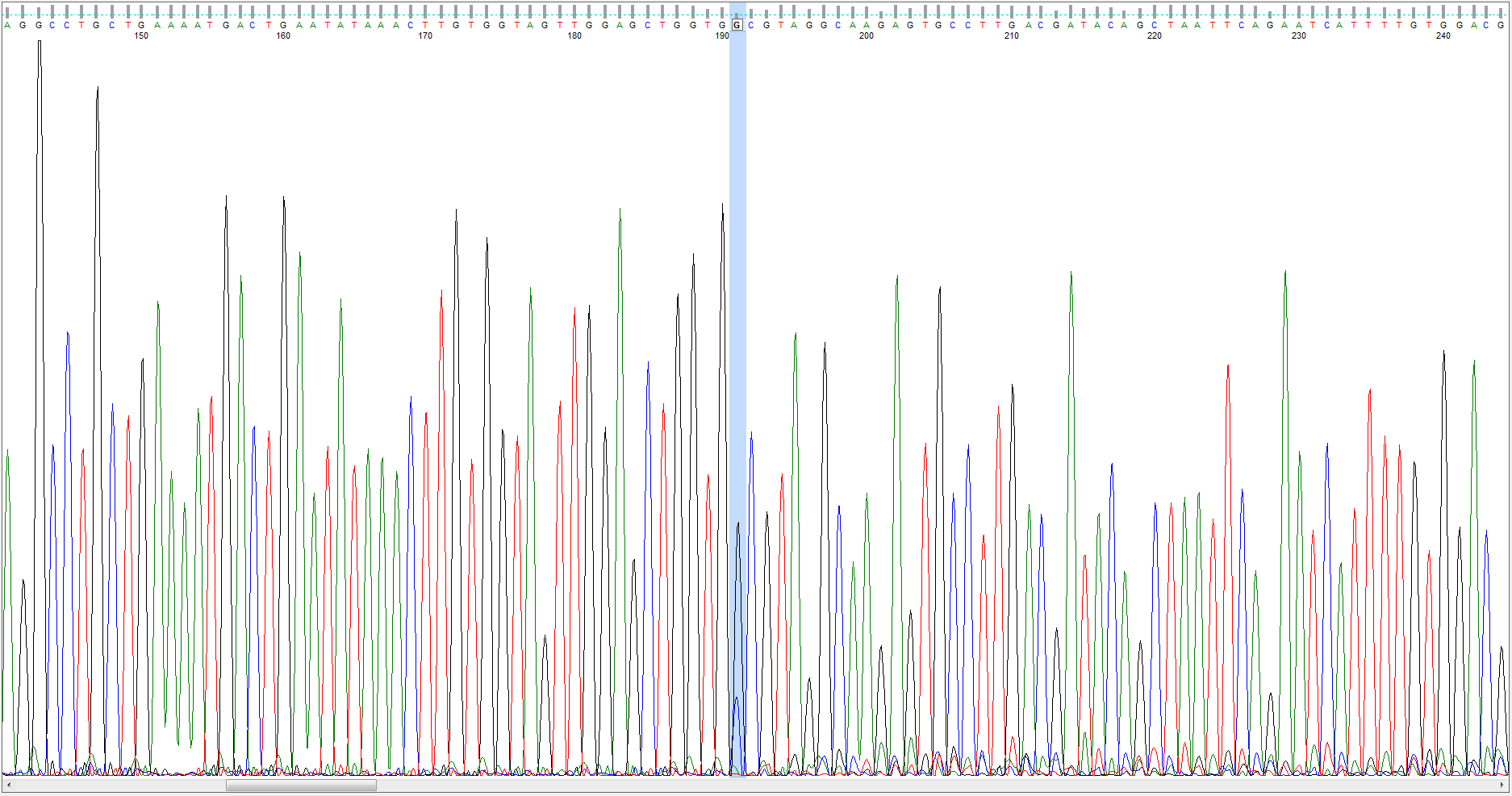


**S11 Fig. The Sanger sequencing results of the genomic DNA from SW48 HT-Cas9 cells.**

Supplement: S11 Fig — (DOCX) [file pone.0144970.s011.docx]

**Figure S12**


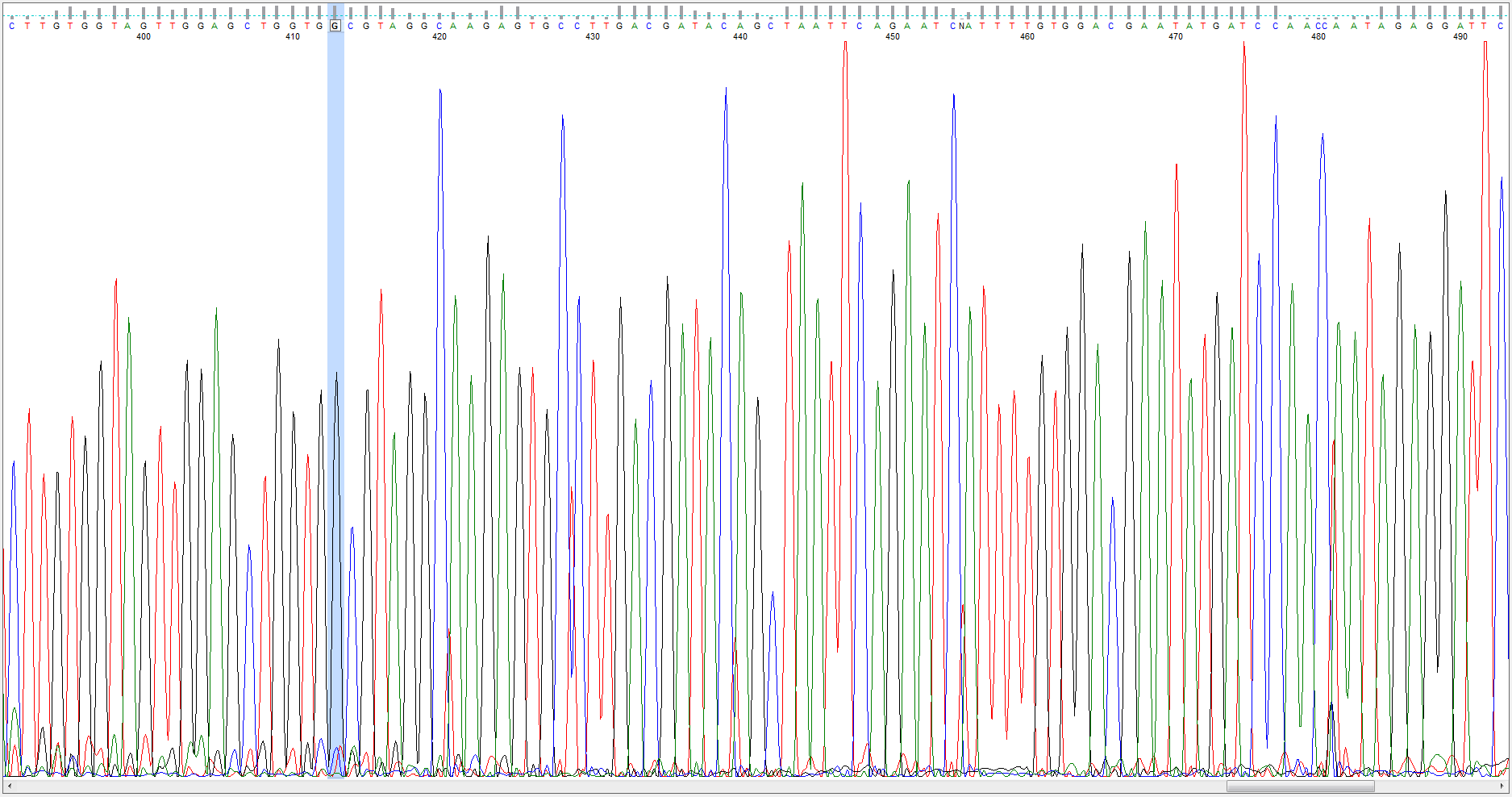


**S12 Fig. The Sanger sequencing results of the mRNA from SW48 HT-Cas9 cells.**

Supplement: S12 Fig — (DOCX) [file pone.0144970.s012.docx]
