# Supplementary material for: Exploiting the CRISPR/Cas9 PAM Constraint for Single-Nucleotide Resolution Interventions
Source: PLoS One. 2016 Jan 20;11(1):e0144970. doi: 10.1371/journal.pone.0144970 (PMC4720446; doi:10.1371/journal.pone.0144970)
Supplement: S8 Fig — (DOCX) [file pone.0144970.s008.docx]

**Figure S8**

**
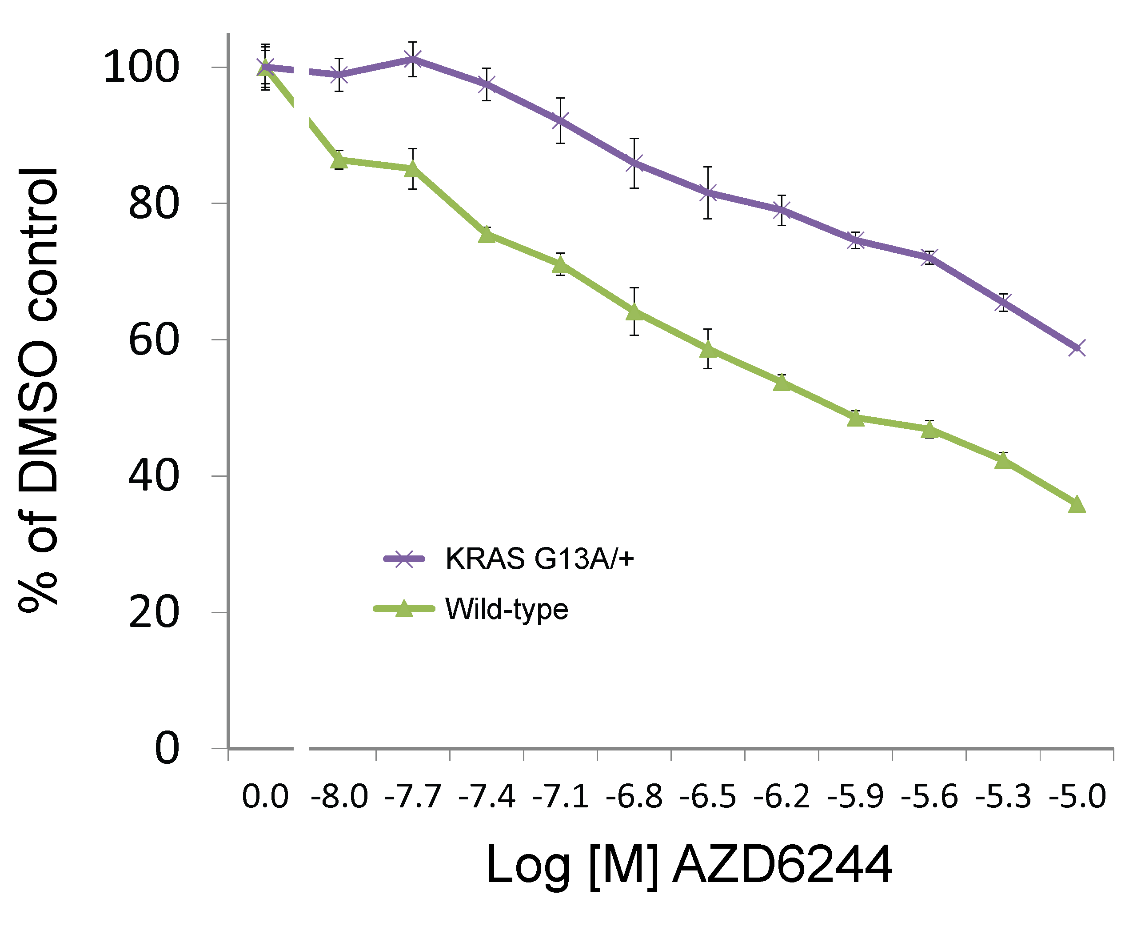
**

**S8 Fig. The KRAS G13A/+ stable SW48 cell line demonstrated increased resistance to AZD6244.** The drug response curves of the KRAS G13A/+ SW48 (HT) cells and their parental wild type (WT) SW48 cells to the MEK inhibitor AZD6244. The KRAS HT stable cell line showed increased resistance to AZD6244.
