# Supplementary material for: Exploiting the CRISPR/Cas9 PAM Constraint for Single-Nucleotide Resolution Interventions
Source: PLoS One. 2016 Jan 20;11(1):e0144970. doi: 10.1371/journal.pone.0144970 (PMC4720446; doi:10.1371/journal.pone.0144970)
Supplement: S13 Fig — (DOCX) [file pone.0144970.s013.docx]

**Figure S13**


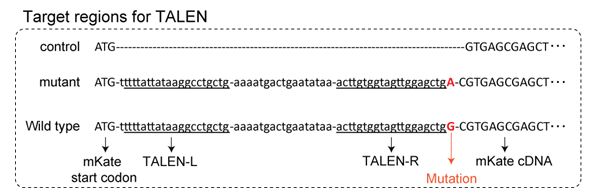


**S13 Fig. Schematic illustration of the fluorescence reporter plasmid for exploring the N0 constraint of TALENs.** The variable regions, which correspond to either KRAS wild type or p.D12G mutant alleles, were shown in the box and cloned after the mKate start codon.
