# Supplementary material for: Exploiting the CRISPR/Cas9 PAM Constraint for Single-Nucleotide Resolution Interventions
Source: PLoS One. 2016 Jan 20;11(1):e0144970. doi: 10.1371/journal.pone.0144970 (PMC4720446; doi:10.1371/journal.pone.0144970)
Supplement: S14 Fig — (DOCX) [file pone.0144970.s014.docx]

**Figure S14**


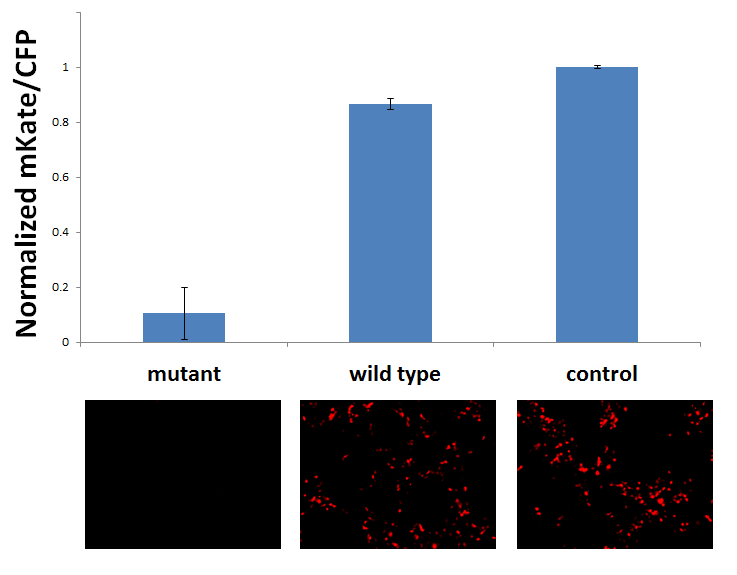


**S14 Fig: TALEN N0 constraint-mediated genome editing of the KRAS p.D12G mutations *in vitro*.** Flow cytometry and fluorescence microcopy results for the normalized mKate expression. Both assays demonstrated that TALENs specifically silenced the mKate carrying the KRAS p.D12G mutant sequence.
