# Supplementary material for: Exploiting the CRISPR/Cas9 PAM Constraint for Single-Nucleotide Resolution Interventions
Source: PLoS One. 2016 Jan 20;11(1):e0144970. doi: 10.1371/journal.pone.0144970 (PMC4720446; doi:10.1371/journal.pone.0144970)
Supplement: S1 Table — (DOCX) [file pone.0144970.s015.docx]

**S1 Table**

| **primer** | **sequence (5'->3')** |
| --- | --- |
| P1 | CACACATTAAAATGTCGTCAAATATTGTT |
| P2 | TCAGATAACTTAACTTTCAGCATAATTATC |
| P3 | CGTCTGCAGTCAACTGGAATTTTCA |
| P4 | ATGACTGAATATAAACTTGTGGTAG |
| P5 | GTCCTCATGTACTGGTCCCTCATTG |
| P6 | CCTGCAGGATCCATGGTGAGCGAGCTGATTAAGGAGAACA |
| P7 | CCTGCAGATATCTTAGACGTTGATCCTGGCGCTGGCG |
| P8 | CAGTACACGCGTCCTGCGTTATCCCCTGATTCTGTGG |
| P9 | CAGTACACGCGTATCGATTACAATTTACGCCTTAAGATACATTG |
| P10 | CCTGCAGGATCCATGGTCAAGGCACTCTTGCCTACGCCCGTGAGCGAGCTGATTAAGGAGAACA |
| P11 | CCTGCAGGATCCATGGTCAAGGCACTCTTGCCTACGGCCGTGAGCGAGCTGATTAAGGAGAACA |
| P12 | TTTCTTGGCTTTATATATCTTGTGGAAAGGACGAAACACCGGTCAAGGCACTCTTGCCTA |
| P13 | GACTAGCCTTATTTTAACTTGCTATTTCTAGCTCTAAAACTAGGCAAGAGTGCCTTGACC |
| P14 | GGAACCAATTCAGTCGACTGGATC |
| P15 | CAGTACATCGATCACACATTAAAATGTCGTCAAATATTGTTC |
| P16 | CAGTACGAGCTCTCAGATAACTTAACTTTCAGCATAATTATC |
| P17 | TAGTTGGAGCTGGTGCCGTAGGCAAGAGTGC |
| P18 | GCACTCTTGCCTACGGCACCAGCTCCAACTA |
| P19 | CAGTACGAGCTCAATGTACCTTGGGTTTCAAGTTATATGTAA |
| P20 | CAGTACGAATTCGCTAAAGTATTAGGACTGCTTAACCCAGGG |
| P21 | CAGTACGAGCTCCTGGGACGGAGGCTTGTTTGCGAG |
| P22 | CAGTACGAGCTCGGGCGACCCGCTCCTAGCAAAGGT |
| P23 | TTTCTTGGCTTTATATATCTTGTGGAAAGGACGAAACACCGGTTATCTGAAATGTACCTT |
| P24 | GACTAGCCTTATTTTAACTTGCTATTTCTAGCTCTAAAACAAGGTACATTTCAGATAACC |
| P25 | CACCGCGTCAAGGCACTCTTGCCTA |
| P26 | AAACTAGGCAAGAGTGCCTTGACGC |
| P27 | GAGGGCCTATTTCCCATGATT |

**S1 Table**: Primers used in this study.
