# Supplementary material for: Exploiting the CRISPR/Cas9 PAM Constraint for Single-Nucleotide Resolution Interventions
Source: PLoS One. 2016 Jan 20;11(1):e0144970. doi: 10.1371/journal.pone.0144970 (PMC4720446; doi:10.1371/journal.pone.0144970)
Supplement: S1 Text — (DOCX) [file pone.0144970.s016.docx]

**Text S1**

**General cloning protocols**

The human codon optimized Cas9 containing nuclear localization signals and an empty gRNA backbone were obtained from Addgene (plasmid #41815 and #41824) [1]. Phusion Hot Start Flex 2X Master Mix (New England Biolabs) or Q5 High-Fidelity 2X Master Mix (New England Biolabs) was used for all polymerase chain reactions (PCR) according to the manufacturer’s protocol. All oligonucleotides were ordered from Sigma-Aldrich and were listed in **S1 Table**. The plasmids were constructed using PCR amplification, restriction digest (all restriction enzymes were ordered from New England Biolabs), and ligation with T4 DNA ligase (New England Biolabs). Gel purification and PCR purification were performed with QIAquick Gel Extraction and PCR Purification kits (Qiagen). Transformations were performed using NEB 5-alpha electrocompetent *Escherichia Coli* (New England Biolabs). The minipreps were performed using QIAprep Spin Miniprep kit (Qiagen). The final plasmids were confirmed by both restriction enzyme digestions and direct Sanger sequencings.

**DNA Constructs**

**PCMV-CFP-TRE-control-mKate**: pTRE3G-BI was purchased from Clontech (catalog number: 631332). PCMV-CFP was purchased from Evrogen (catalog number: FP112). The mKate-PEST sequence was PCR amplified from PCMV-mKate-PEST-PCMV-YFP-PEST (unpublished results) using primers P6 and P7 and cloned into the pTRE3G-BI vector using BamHI and ECoRV sites. Next, the PCMV-CFP sequence was PCR amplified from PCMV-CFP using primers P8 and P9 and cloned into the TRE-control-mKate plasmid using MluI site.

**PCMV-CFP-TRE-wild type-mKate**: pTRE3G-BI was purchased from Clontech (catalog number: 631332). PCMV-CFP was purchased from Evrogen (catalog number: FP112). The modified mKate-PEST sequence was PCR amplified from PCMV-mKate-PEST-PCMV-YFP-PEST (unpublished results) using primers P10 and P7 and cloned into the pTRE3G-BI vector using BamHI and ECoRV sites. Next, the PCMV-CFP sequence was PCR amplified from PCMV-CFP using primers P8 and P9 and cloned into the TRE-wild type-mKate plasmid using MluI site.

**PCMV-CFP-TRE-mutant-mKate**: pTRE3G-BI was purchased from Clontech (catalog number: 631332). PCMV-CFP was purchased from Evrogen (catalog number: FP112). The modified mKate-PEST sequence was PCR amplified from PCMV-mKate-PEST-PCMV-YFP-PEST (unpublished results) using primers P11 and P7 and cloned into the pTRE3G-BI vector using BamHI and EcoRV sites. Next, the PCMV-CFP sequence was PCR amplified from PCMV-CFP using primers P8 and P9 and cloned into the TRE-mutant-mKate plasmid using MluI site.

**U6-KRAS G13A-targeting gRNA**: The U6-KRAS G13A-targeting gRNA construct was prepared according to the manufacturer’s protocol (1). Briefly, primers P12 and P13 were annealed, extended and then cloned into the U6 gRNA vector using AflII site. The construct was confirmed by the Sanger sequencing using the primer P14.

**Donor plasmid for generating the KRAS G13A/+ mutant cell lines**: The genomic DNA was isolated from wild type SW48 cells using DNeasy Blood&Tissue Kit (Qiagen). The left arm was amplified with primers P15 and P16 using the SW48 genomic DNA as the template, and then cloned into PEF-1 vector using ClaI and SacI sites. Next, the KRAS 13^th^ codon point mutation (GGC->GCC) was introduced by using the QuickChange II XL Site-Directed Mutagenesis Kit (Agilent Technolgies) and primers P17 and P18. Next, the right arm was amplified with primers P19 and P20 using the SW48 genomic DNA as the template, and then cloned into the above plasmid using SacI and EcoRI sites. Finally, the puromycin resistance gene cassette sequence was amplified from PCMV-puromycin resistance gene (unpublished results) using primers P21 and P22, and then cloned into the above plasmid using AgeI and EcoRI sites.

**gRNA construct for generating the KRAS G13A/+ mutant cell lines**: The gRNA sequence (5’-AGTTATCTGAAATGTACCTT-3’) was designed using CRISPR design (<http://crispr.mit.edu/>). The U6-gRNA construct was prepared according to the manufacturer’s protocol. Briefly, primers P23 and P24 were annealed, extended and then cloned into the U6 gRNA vector using AflII site. The construct was confirmed by the Sanger sequencing using the primer P14.

**References**

1. Mali P, Yang L, Esvelt KM, Aach J, Guell M, DiCarlo JE, Norville JE, Church GM. RNA-guided human genome engineering via Cas9. *Science.* 2013 Feb 15; 339(6121): 823-826. PMCID: PMC3712628.
